# Efficacy and Safety of High-Power Short-Duration Radiofrequency Catheter Ablation of Atrial Fibrillation

**DOI:** 10.3389/fcvm.2021.709585

**Published:** 2021-10-07

**Authors:** Je-Wook Park, Song-Yi Yang, Min Kim, Hee Tae Yu, Tae-Hoon Kim, Jae-Sun Uhm, Boyoung Joung, Moon-Hyoung Lee, Hui-Nam Pak

**Affiliations:** Division of Cardiology, Yonsei University Health System, Seoul, South Korea

**Keywords:** atrial fibrillation, catheter ablation, radiofrequency, power, duration (time), efficacy and safety

## Abstract

**Introduction:** Whereas, high-power short-duration (HPSD) radiofrequency (RF) ablation is generally used in atrial fibrillation (AF) catheter ablation (CA), its efficacy, safety, and influence on autonomic function have not been well established in a large population. This study compared HPSD-AFCA and conventional power (ConvP)-AFCA in propensity score matched-population.

**Methods:** In 3,045 consecutive patients who underwent AFCA, this study included 1,260 patients (73.9% male, 59 ± 10 years old, 58.2% paroxysmal type) after propensity score matching: 315 in 50~60W HPSD group vs. 945 in the ConvP group. This study investigated the procedural factors, complication rate, rhythm status, and 3-month heart rate variability (HRV) between the two groups and subgroups.

**Results:** Procedure time was considerably short in the HPSD group (135 min in HPSD vs. 181 min in ConvP, *p* < 0.001) compared to ConvP group, but there was no significant difference in the complication rate (2.9% in HPSD vs. 3.7% in ConvP, *p* = 0.477) and the 3-month HRV between the two groups. At the one-year follow-up, there was no significant difference in rhythm outcomes between the two groups (Overall, Log-rank *p* = 0.885; anti-arrhythmic drug free, Log-rank *p* = 0.673). These efficacy and safety outcomes were consistently similar irrespective of the AF type or ablation lesion set. The Cox regression analysis showed that the left atrium volume index estimated by computed tomography (HR 1.01 [1.00–1.02]), *p* = 0.003) and extra-pulmonary vein triggers (HR 1.59 [1.03–2.44], *p* = 0.036) were independently associated with one-year clinical recurrence, whereas the HPSD ablation was not (HR 1.03 [0.73–1.44], *p* = 0.887).

**Conclusion:** HPSD-AFCA notably reduced the procedure time with similar rhythm outcomes, complication rate, and influence on autonomic function as ConvP-AFCA, irrespective of the AF type or ablation lesion set.

## Introduction

Atrial fibrillation catheter ablation (AFCA) is an efficient modality for maintaining sinus rhythm and has beneficial effect on symptoms and quality of life in AF patients (1). Recent studies and guidelines showed the beneficial effect of AFCA for mortality and hospitalization in AF patients with reduced left ventricular function (1, 2). However, there is still a controversial issue for other clinical benefits including overall mortality and stroke (3). Despite these clinical benefits, the long-term AF recurrence rate after AFCA procedures remains unsatisfactory (4, 5). Continuous recurrence after the procedure is a reason why AF itself is a chronic progressive disease, but it is also a limitation of the ablation procedures currently used. The pulmonary vein isolation (PVI) maintenance rate has been reported to be 5–40% after radiofrequency catheter ablation and 40~60% after cryoballoon PVI (6–8). In recent years, proper and standardized ablation lesion formation can be indicated by contact force monitoring, the ablation index, or the lesion index for a more effective AFCA (9). High-power short-duration (HPSD) radiofrequency (RF) ablation is suitable for wide and contiguous lesions because it uses resistive heating rather than RF conductive heating (10). In particular, since the atrial wall thickness is <4 mm at most, the atrial wall is a good target for HPSD ablation, which forms wide and thin RF lesions (11, 12). Nevertheless, data comparing the HPSD-AF catheter ablation (AFCA) and conventional RF power AFCA are relatively limited. In this study, we compared the outcome of HPSD-AFCA and AFCA using conventional RF energy with an irrigated tip ablation catheter after propensity matching of single-center AFCA cohort data with a consistent ablation protocol. Unlike previous comparative studies, we compared the differences in the AF type, ablation lesion set, and 3rd-month heart rate variability (HRV), which reflects cardiac autonomic nerve modulation effects in this study.

## Methods

### Study Population

**Figure 1:**
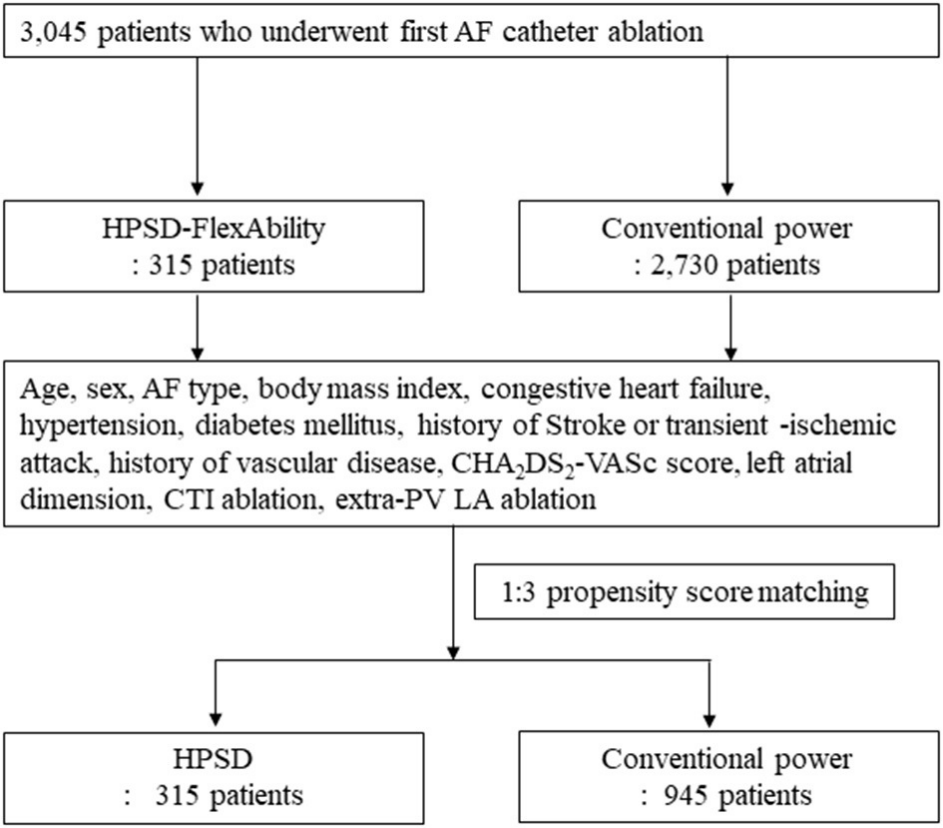
Study flow chart, HPSD, high power short duration; AF, atrial fibrillation.

This study protocol adhered to the principles of the Declaration of Helsinki and was approved by the Institutional Review Board of the Yonsei University Health System. All patients provided written informed consent for inclusion in the Yonsei AF Ablation Cohort Database (ClinicalTrials.gov Identifier: NCT02138695). Between March 2009 and April 2020, we investigated 3,045 patients in the Yonsei AF Ablation Cohort Database who underwent a *de novo* AFCA. Patients were categorized into two groups: HPSD-RF and conventional power RF. In this study, the conventional power RF group was enrolled from 2009 to 2020. And HPSD-RF group was enrolled from 2018 to 2020. This study conformed to the following exclusion criteria: (1) AF with rheumatic valvular disease, (2) significant structural heart disease other than left ventricular hypertrophy, and (3) a history of prior AF ablation or cardiac surgery. After 1:3 propensity score matching, 1,260 patients were analyzed: 315 patients in the HPSD ablation group and 945 in the conventional power AFCA group. The patient flow chart of this study is presented in Figure 1.

All patients underwent three-dimensional (3D) spiral computer tomography (CT) (64 Channel, Light Speed Volume CT, Philips, Brilliance 63, Amsterdam, The Netherlands) to visually identify the anatomy of the LA and pulmonary veins. An oral anticoagulation therapy was performed in all patients using vitamin K antagonists or non-vitamin K oral anticoagulants at least 1 month before and 2 months after the procedure. We stopped all antiarrhythmic drugs for at least five half-lives and amiodarone at least 4 weeks before the procedure.

### Electrophysiologic Mapping and Radiofrequency Catheter Ablation

Intracardiac electrograms were recorded using the Prucka CardioLab™ Electrophysiology system (General Electric Medical Systems, Inc., Milwaukee, WI, USA), and we conducted the AFCA in all patients by a 3D electroanatomical mapping system (NavX; St. Jude Medical or CARTO; Biosense Webster, USA.) merged with 3D spiral CT. We performed trans-septal punctures and then obtained multi-view pulmonary venograms. Details of the AFCA technique and strategy have been reported in our previous studies (13). We performed systemic anticoagulation using intravenous heparin to achieve an activated clotting time of 350–400 s during the procedure.

The details of the RFCA technique and strategy were described in our previous studies (14). Contact force sensing catheters were used in 9.3% (117 patients) of the overall study population only in the conventional power group. For the conventional power RF ablation, we used an open-irrigated, 3.5-mm tip deflectable catheter (Celsius and Smart-Touch; Johnson & Johnson, Inc., Diamond Bar, CA; Coolflex and FlexAbility; St. Jude Medical, Inc., Minnetonka, MN) with an RF power of 25–35 W (power-controlled mode, 45°C). We used a 30–35 W ablation for the anterior side of the LA and PVs and a 20–25 W ablation for the posterior side of the LA and PVs. When we used contact force sensing catheters, the target of ablation index was 400–450 at the anterior side of the LA and PVs and 300–350 at the posterior side of the LA and PVs. For the HPSD-RF ablation, we used a FlexAbility catheter (St. Jude Medical, Inc., Minnetonka, MN) without contact force monitoring. In 242 patients (77%) of the HPSD group, we used a 50 W ablation with 10~15 s for the anterior side of the LA and PVs and 40 W ablation with a reduced ablation time of <10 s for the posterior side of the LA and PVs for CPVI. In 73 patients (23%) of the HPSD group, we used a 60 W ablation for the anterior side of the LA and PVs and 50 W for the posterior side of the LA and PVs for CPVI. We monitored the esophageal temperature in all patients who underwent HPSD-AFCA. We started to monitor the esophageal temperature from 2019, so the majority of conventional power groups were not monitored esophageal temperature. If the esophageal temperature rose over 38.4°C, we moved the ablation site away from the esophagus until the temperature came back to normal. To minimize any esophageal temperature rise, the operators ablated the esophagus contact area by taking a step back and forth with spacing rather than with contiguous ablation lesions. When the esophageal temperature rose in this area, we moved the ablation catheter to another place away from the esophagus to continue RF ablation, and then returned to fill the gap when the temperature dropped.

All patients underwent a CPVI. For a CPVI, circumferential lesions were continuously created at the level of the LA antrum encircling the right and left PVs. The purpose of the CPVI was to achieve the electric isolation of the PV potentials and bidirectional block of the PVs. We confirmed the CPVI during an isoproterenol infusion after a waiting time of 30 min. A cavotricuspid isthmus (CTI) block procedure was performed in the majority of the patients (98.7%) during the AFCA except the patients with AV conduction disease. We added an additional linear ablation including a roof line, posterior-inferior line (posterior box lesion), or anterior line, especially in patients with persistent AF. An ablation for mitral isthmus line, right atrium, and complex fractionated electrogram was conducted in a minority of the patients depending on operator's discretion. We defined extra-PV LA ablation as any additional linear ablation including roof line, posterior-inferior line, anterior line, or mitral isthmus line after CPVI.

We finished the *de novo* procedure unless the AF was promptly recurred within 10 min after cardioversion with an isoproterenol infusion (5~20 μg/min depending on ß-blocker use and target sinus heart rate of 120 bpm). In the case of mappable AF triggers or premature atrial beats, extra-PV foci were carefully mapped and ablated to the greatest extent possible.

### Holter Monitor Recordings and the Heart Rate Variability Analysis

The HRV was evaluated *via* 24-h Holter monitor recordings obtained during the pre- and post-ablation periods at 3, 12, and 24 months with a GE Marquette MARS 8000 Holter analyzer (General Electric Medical System, Inc.). Following recognizing each QRS complex, the numerical series of RR intervals were measured. Only high-quality recordings were selected for the analysis. All recordings were digitized and reviewed by an experienced operator. Premature ventricular beats, premature atrial beats, and electrical artifacts were excluded from the analysis. The HRV parameters were utilized to present autonomic activity depending on the previously published guidelines (15). The ganglion plexus modification around the PV (16) and the third fat pad (14, 17) could be assessed by the HRV and associated with AF recurrence after the AFCA. Because the ganglionate plexi are located in the epicardial area (18), we assumed that transmural lesion formation might be an important factor for AF recurrence and related to the HRV. The mean heart rate and following time-domain HRV parameters were analyzed: mean RR interval (mean NN interval), the standard deviation of the NN intervals, the standard deviation of the 5 min means of the NN intervals, and root mean square of the differences between successive NN intervals (rMSSD). The following parameters were calculated: very-low-frequency components (VLF; 0.040 Hz), low-frequency components (LF; 0.040–0.150 Hz), high-frequency components (HF; 0.150–0.400 Hz), and the LF/HF ratio. The high-frequency components and rMSSD represented parasympathetic nervous activity, and the LF and LF/HF ratio represented the sympathetic nervous activity and sympathovagal balance, respectively.

### Post-ablation Management and Follow-Up

We discharged patients without antiarrhythmic drugs (AADs) except for those who had recurrent extra-PV triggers after the AFCA procedure, symptomatic frequent atrial premature beats, non-sustained atrial tachycardia, or an early recurrence of AF on telemetry during the admission period (27.2%). We scheduled to regularly check the patients in the outpatient clinic at 1, 3, 6, and 12 months, and then every 6 months thereafter or whenever the patients experienced symptoms after RFCA. We obtained an ECG in all patients every visit and 24-h Holter recordings at 3 and 6 months and every 6 months thereafter, depending on the 2012 Heart Rhythm Society/European Heart Rhythm Association/European Cardiac Arrhythmia Society Expert Consensus Statement guidelines. We examined the patients who experienced symptoms of palpitations representing an arrhythmia recurrence using Holter monitor or event monitor recordings. A researcher who was independent to the study group assignment conducted a Holter analysis and adjudication. AF recurrence was defined as any episode of AF or atrial tachycardia (AT) at least 30 s in duration. Any ECG documentation of AF recurrence within a 3-month after the procedure was considered as an early recurrence, and an AF recurrence >3 months after the procedure was considered as a clinical recurrence. The primary endpoint was the freedom from documented episodes of AF or AT lasting longer than 30 s and occurring after a 3-month blanking period within a year after a single ablation procedure, with or without the use of AADs.

### Statistical Analysis

Continuous variables are reported as the mean ± standard deviation (SD), and categorical variables are reported as the numbers (percentage). Continuous variables were analyzed by independent *t*-test, whereas categorical variables were analyzed by a Chi-square test or Fisher's exact test. We performed propensity-score matching using the nearest neighbor method without a replacement and a caliper at a 1: 3 ratio of the HPSD and the conventional power groups. The following variables including age, sex, AF type, body mass index, CHA_2_DS_2_-VASc score and comorbidities, LA dimension, cavotricuspid (CTI) ablation, and extra-PV LA ablation were included in the propensity-score matching. The standardized mean differences of all adjusted variables were under 0.1 after propensity-score matching (Supplementary Table 1). A Kaplan–Meier analysis with a log-rank test was used to compare the freedom from AF recurrence between two groups. A multivariable Cox proportional hazards regression analysis without any stepwise methods was used to investigate any predictors associated with 1-year AF recurrence. The variables with *p* ≤0.2 in the univariate Cox regression analysis and age and sex were included in the multivariate Cox regression analysis. A linear regression analysis was performed to investigate the variables which affected the procedure and ablation times. The variables with *p* ≤0.2 in the univariate linear regression analysis were included in the multivariate linear regression analysis. A two-sided *P* < 0.05 was considered to indicate statistical significance. The statistical analyses were conducted using the Statistical Package for Social Sciences version 23.0 (SPSS Inc, Chicago, IL, USA) and R version 3.4.4 (R Foundation for Statistical Computing, Vienna, Austria).

## Results

### Patient Characteristics and Procedural Results

We compared 315 patients in the HPSD group and 945 in the conventional power group after 1:3 propensity score matching (Figure 1). Table 1 showed no significant differences in age, the proportion of male sex, AF type, and comorbidities between the two groups. The echocardiographic and CT parameters did not significantly differ between the two groups except the left ventricular ejection fraction (*p* = 0.002, Table 1).

**Table 1:** Baseline characteristics in propensity-score matched population.

	**Overall**	**HPSD**	**Conventional RFA**	***p*-value**
	**(*n* = 1,260)**	**(*n* = 315)**	**(*n* = 945)**	
Age, years	59 ± 10	59 ± 11	59 ± 10	0.875
Male, *n* (%)	931 (73.9)	232 (73.7)	699 (74.0)	0.912
Paroxysmal AF, *n* (%)	733 (58.2)	180 (57.1)	553 (58.5)	0.668
Body mass index, kg/m^2^	25.3 ± 3.3	25.3 ± 3.2	25.3 ± 3.4	0.730
Comorbidities, *n* (%)				
Congestive heart failure	272 (21.6)	70 (22.2)	202 (21.4)	0.752
Hypertension	606 (48.1)	151 (47.9)	455 (48.1)	0.948
Diabetes mellitus	206 (16.3)	53 (16.8)	153 (16.2)	0.792
Stroke/TIA	153 (12.1)	38 (12.1)	115 (12.2)	0.960
Vascular disease	61 (4.8)	14 (4.4)	47 (5.0)	0.705
CHA_2_DS_2_-VASc score	1.8 ± 1.5	1.8 ± 1.6	1.8 ± 1.5	0.897
Echocardiographic parameters				
LA dimension, mm	42.6 ± 6.3	42.8 ± 6.6	42.5 ± 6.2	0.568
LV ejection fraction, %	61.9 ± 9.3	63.2 ± 8.2	61.5 ± 9.6	0.002
E/Em (*n* = 1,204)	10.4 ± 4.3	10.5 ± 4.5	10.4 ± 4.3	0.746
LVEDD, mm (*n* = 1,257)	50.5 ± 4.7	50.5 ± 4.4	50.5 ± 4.8	0.973
CT volume index, cm^3^/m^2^ (*n* = 1,232)	87.8 ± 26.7	88.1 ± 25.4	87.7 ± 7.1	0.796

*HPSD, High power short duration; RFA, radiofrequency ablation; AF, atrial fibrillation; TIA, transient ischemic attack; LA, left atrium; LV, left ventricle; E/Em, mitral inflow velocity/mitral annulus tissue velocity; LVEDD, LV end-diastolic diameter; CT, computed tomography*.

The procedural results are presented in Table 2. The procedure time (*p* < 0.001) and ablation time (*p* < 0.001) were remarkably shorter in the HPSD group than the conventional power group. The proportion of CTI ablation (*p* > 0.999), empirical extra-PV LA ablation (*p* = 0.854), and post-ablation extra-PV triggers (*p* = 0.219) did not show significant difference between the two groups. The overall complication rates were similar between the two groups (2.9% in the HPSD group vs. 3.7% in the conventional power group, *p* = 0.477, Table 2).

**Table 2:** Ablation characteristics in propensity-score matched population.

	**Overall**	**HPSD**	**Conventional RFA**	***p-*value**
	**(*n* = 1,260)**	**(*n* = 315)**	**(*n* = 945)**	
Procedure time, minutes	169.3 ± 52.9	135.0 ± 30.3	180.7 ± 53.8	<0.001
Ablation time, seconds	4260.1 ± 1648.6	2756.4 ± 743.2	4765.7 ± 1559.0	<0.001
60W/50W in anterior side of LA and PVs, *n* (%)	73 (6)/242 (19)	73 (23)/242 (77)^*^	0	
Ablation lesion set, *n* (%)				
CPVI ablation	1,260 (100)	315 (100)	945 (100)	NA
CTI ablation	1,243 (98.7)	311 (98.7)	932 (98.6)	>0.999
Extra-PV LA ablation	337 (26.7)	83 (26.3)	254 (26.9)	0.854
POBI	240 (19.0)	41 (13.0)	199 (21.1)	
Anterior line	217 (17.2)	19 (6.0)	198 (21.0)	
Mitral isthmus line	53 (4.2)	16 (5.1)	37 (3.9)	
Extra-PV trigger, *n (%)*	111/893 (12.4)	39/269 (14.5)	72/624 (11.5)	0.219
Complications, *n (%)*	44 (3.5)	9 (2.9)	35 (3.7)	0.477
Type of complications, *n (%)*^†^				
AE fistula	1 (0.08)	0	1 (0.11)	
Hemopericardium	19 (1.51)	6 (1.90)	13 (1.38)	
Stroke or TIA	1 (0.08)	0	1 (0.11)	
Others^‡^	25 (1.98)	4 (1.27)	21 (2.22)	

*HPSD, high power short duration; RFA, radiofrequency ablation; CPVI, circumferential pulmonary vein isolation; NA, not available; CTI, cavotricuspid isthmus; PV, pulmonary vein; LA, left atrium; POBI, posterior box isolation; AE, atrio-esophageal; TIA, transient ischemic attack*.

* *The rate represented the proportion of the patients who had each power (50W or 60W) on the anterior side of LA and PVs. In 242 patients (77%) of the HPSD group, a 50W ablation was used for the anterior side of the LA and PVs. In 73 patients (23%) of the HPSD group, a 60W ablation was used for the anterior side of the LA and PVs*.

† *Type of complications were not mutually exclusive*.

‡ *Others include pericarditis, complete atrioventricular block, sinus node dysfunction, arteriovenous fistula, puncture site bleeding or hematoma, other bleeding, phrenic nerve palsy, and any shock*.

### Rhythm Outcomes

**Figure 2:**
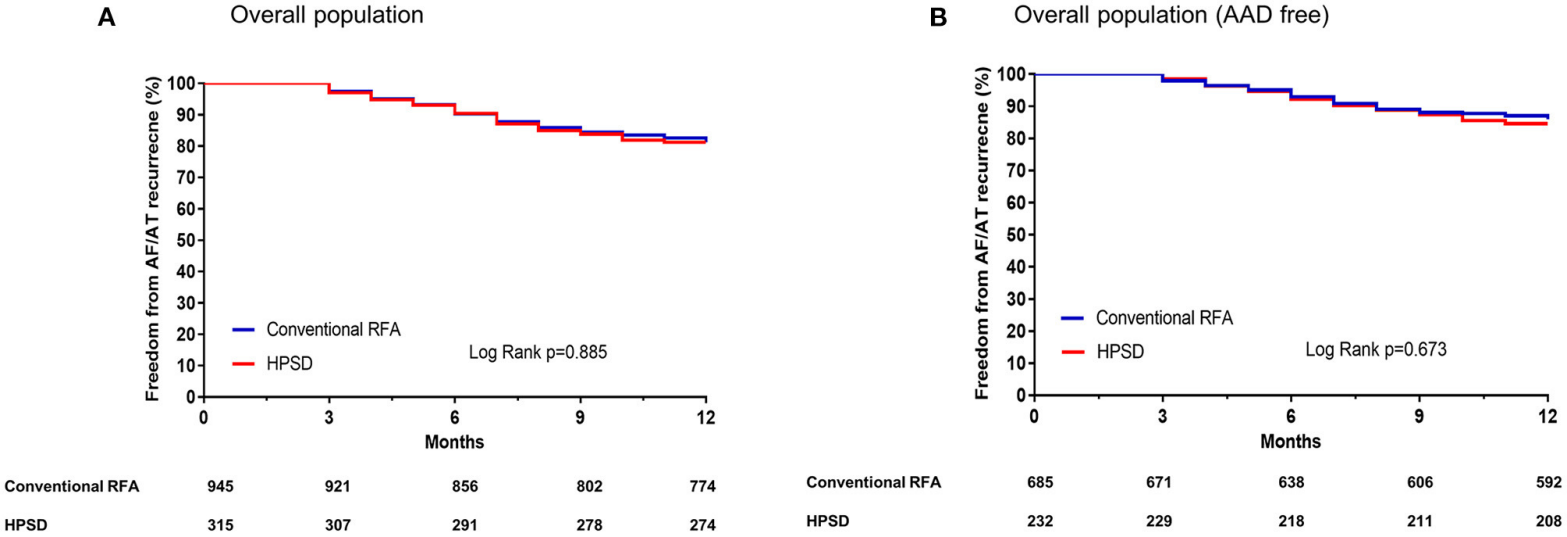
Kaplan–Meier curves for the atrial fibrillation (AF) recurrence-free survival rates in the overall population **(A)** and in the population without antiarrhythmic drugs [AAD, **(B)**]. HPSD, high-power short-duration radiofrequency ablation; RFA, radiofrequency ablation.

Because the regular rhythm follow-up durations differed between the two groups, we evaluated the AF recurrence within 12 months after a single AFCA procedure. In Kaplan–Meier analysis, the one-year clinical recurrence rate was similar between the two groups (overall, Log rank *p* = 0.885; AAD free, Log rank *p* = 0.673, Figure 2). And AT recurrence rate (overall 14.6% in the HPSD group vs. 29.4% in conventional power group *p* = 0.054) and cardioversion rate after recurrence (22% in the HPSD group vs. 32.2% in the conventional power group, *p* = 0.201) did not significantly differ between the HPSD group and the conventional power group within a year (Table 3). In the multivariate Cox regression analysis for clinical recurrence within a year, the CT-measured LA volume index (hazard ratio [HR] 1.01 [1.00–1.02], *p* = 0.003), AADs at discharge (HR 2.69 [1.84–3.93], *p* < 0.001), and extra-PV triggers (HR 1.59 [1.03–2.44], *p* = 0.036) were independently associated, but the HPSD ablation was not (Table 4).

**Table 3:** Clinical rhythm outcomes in propensity-score matched population.

	**Overall**	**HPSD**	**Conventional RFA**	***p*-value**
	**(*n* = 1,260)**	**(*n* = 315)**	**(*n* = 945)**	
Post-ablation medication				
ACEi, or ARB, *n* (%)	466 (37.0)	112 (35.6)	354 (37.5)	0.536
Beta blocker, *n* (%)	506 (40.2)	137 (43.5)	369 (39.1)	0.168
Statin, *n* (%)	424 (33.7)	149 (47.3)	275 (29.1)	**<0.001**
AADs at discharge, *n* (%)	343 (27.2)	83 (26.3)	260 (27.5)	0.688
Early recurrence, *n* (%)	413 (32.8)	99 (31.4)	314 (33.2)	0.556
AT recurrence, *n* (%)	56/211 (26.5)	6/41 (14.6)	50/170 (29.4)	0.054
Cardioversion after recurrence, *n* (%)	64/212 (30.2)	9/41 (22.0)	55/171 (32.2)	0.201
HRV (post AFCA 3months) (*n* = 838)				
Mean heart rate	72 ± 12	74 ± 12	72 ± 11	**0.019**
HF	7.7 ± 7.1	7.4 ± 6.7	7.8 ± 7.2	0.510
rMSSD	19.9 ± 15.4	18.7 ± 13.1	20.2 ± 15.9	0.255
LF	9.3 ± 10.6	9.1 ± 10.4	9.3 ± 10.7	0.863
LF/HF	1.11 ± 0.51	1.11 ± 0.53	1.11 ± 0.5	0.946
HRV (post AFCA 1year) (*n* = 714)				
Mean heart rate	72 ± 11	71 ± 11	72 ± 11	0.473
HF	8.1 ± 7.2	8.4 ± 8.3	8.1 ± 7.1	0.686
rMSSD	21.3 ± 16.6	20.1 ± 14.7	21.5 ± 16.9	0.445
LF	10.9 ± 11.4	11.0 ± 11.7	10.9 ± 11.3	0.926
LF/HF	1.32 ± 0.53	1.31 ± 0.49	1.32 ± 0.53	0.829

*HPSD, high power short duration; RFA, radiofrequency ablation; ACEi, angiotensin-converting enzyme inhibitor; ARB, angiotensin receptor blocker; AAD, antiarrhythmic drugs; AT, atrial tachyarrhythmia; HRV, heart rate variabilities; AFCA, atrial fibrillation catheter ablation; HF, high-frequency components; rMSSD, root-mean square of differences between successive NN intervals; LF, low-frequency components. The bold values mean statistical significance such as p-value < 0.05*.

**Table 4:** Cox regression analysis for 1-year clinical recurrence.

	**Univariate**		**Multivariate**	
	**HR (95% CI)**	***p*-value**	**HR (95% CI)**	***p*-value**
Age, years	0.99 (0.98–1.01)	0.328	0.99 (0.97–1.01)	0.207
Male	0.99 (0.73–1.34)	0.923	0.92 (0.62–1.35)	0.653
Paroxysmal AF	0.77 (0.59–1.00)	0.052	0.90 (0.60–1.35)	0.597
Body mass index	1.01 (0.97–1.05)	0.703		
Congestive heart failure	1.29 (0.95–1.75)	0.107	1.30 (0.87–1.94)	0.208
Hypertension	0.94 (0.72–1.24)	0.668		
Diabetes mellitus	0.89 (0.61–1.30)	0.535		
Stroke/TIA	1.26 (0.86–1.85)	0.230		
Vascular disease	1.06 (0.56-2.00)	0.863		
CHA_2_DS_2_-VASc score	1.02 (0.93–1.11)	0.748		
LA dimension, mm	1.04 (1.02–1.07)	**<0.001**	1.00 (0.96–1.03)	0.782
LV ejection fraction	1.00 (0.98–1.01)	0.856		
E/Em	1.00 (0.97–1.03)	0.922		
LVEDD	1.01 (0.98–1.04)	0.497		
CT volume index	1.01 (1.01–1.02)	**<0.001**	1.01 (1.00–1.02)	**0.003**
CTI ablation	0.86 (0.28-2.69)	0.798		
Extra-PV LA ablation	1.38 (1.04–1.84)	**0.028**	1.04 (0.69–1.57)	0.845
POBI	1.12 (0.80–1.55)	0.519		
Anterior line	1.22 (0.88–1.69)	0.241		
AADs at discharge	2.34 (1.78–3.07)	**<0.001**	2.69 (1.84–3.93)	**<0.001**
Extra-PV triggers	2.13 (1.41–3.22)	**<0.001**	1.59 (1.03–2.44)	**0.036**
HPSD ablation	1.03 (0.73–1.44)	0.887		

*HR, hazard ratio; AF, atrial fibrillation; TIA, transient ischemic attack; LA, left atrium; LV; left ventricle; E/Em, mitral inflow velocity/mitral annulus tissue velocity; LVEDD, left ventricular end diastolic dimension; CT, computed tomography; CTI, cavotricuspid isthmus; PV, pulmonary vein; POBI, posterior box isolation; AAD, antiarrhythmic drugs; HPSD, high power short duration. The bold values mean statistical significance such as p-value < 0.05*.

**Figure 3:**
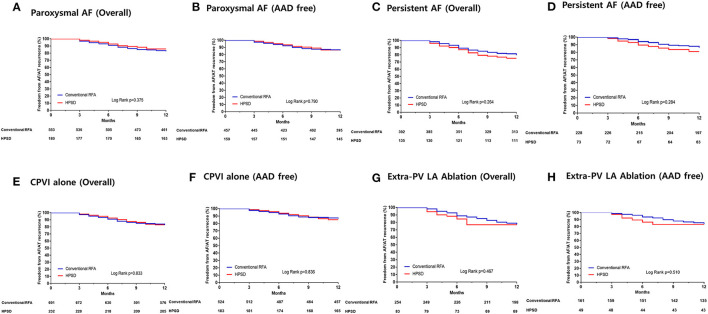
Kaplan–Meier curves for the AF recurrence-free survival rates in the patients with paroxysmal AF **(A,B)**, persistent AF **(C,D)**, who underwent circumferential pulmonary vein isolation (CPVI) alone **(E,F)**, and those who underwent additional extra-pulmonary vein (PV) left atrial (LA) ablations **(G,H)**. HPSD, high-power short-duration radiofrequency ablation; RFA, radiofrequency ablation.

In the subgroup analyses, the 1-year clinical recurrence rates were similar between the HPSD and conventional power groups regardless of the AF type or ablation lesion sets: paroxysmal AF (Figures 3A,B), persistent AF (Figures 3C,D), CPVI alone (Figures 3E,F), and additional extra-PV LA ablation (Figures 3G,H).

### Change in the HRV Parameters After Catheter Ablation

Because cardiac autonomic nerve ganglionate plexi are located in the subepicardial layer, we evaluated the 3rd-month HRV to indirectly assess the transmural ablation lesion formation. Among 1,260 overall patients, 838 patients (168 in the HPSD group and 670 in the conventional power group) had analyzable 3rd-month HRV data. Table 3 showed no statistically significant difference in the HF domain (*p* = 0.510), rMSSD (*p* = 0.255), LF domain (*p* = 0.863), or LF/HF ratio (*p* = 0.946) between the two groups.

### Efficacy in Procedure and Ablation Time

**Figure 4:**
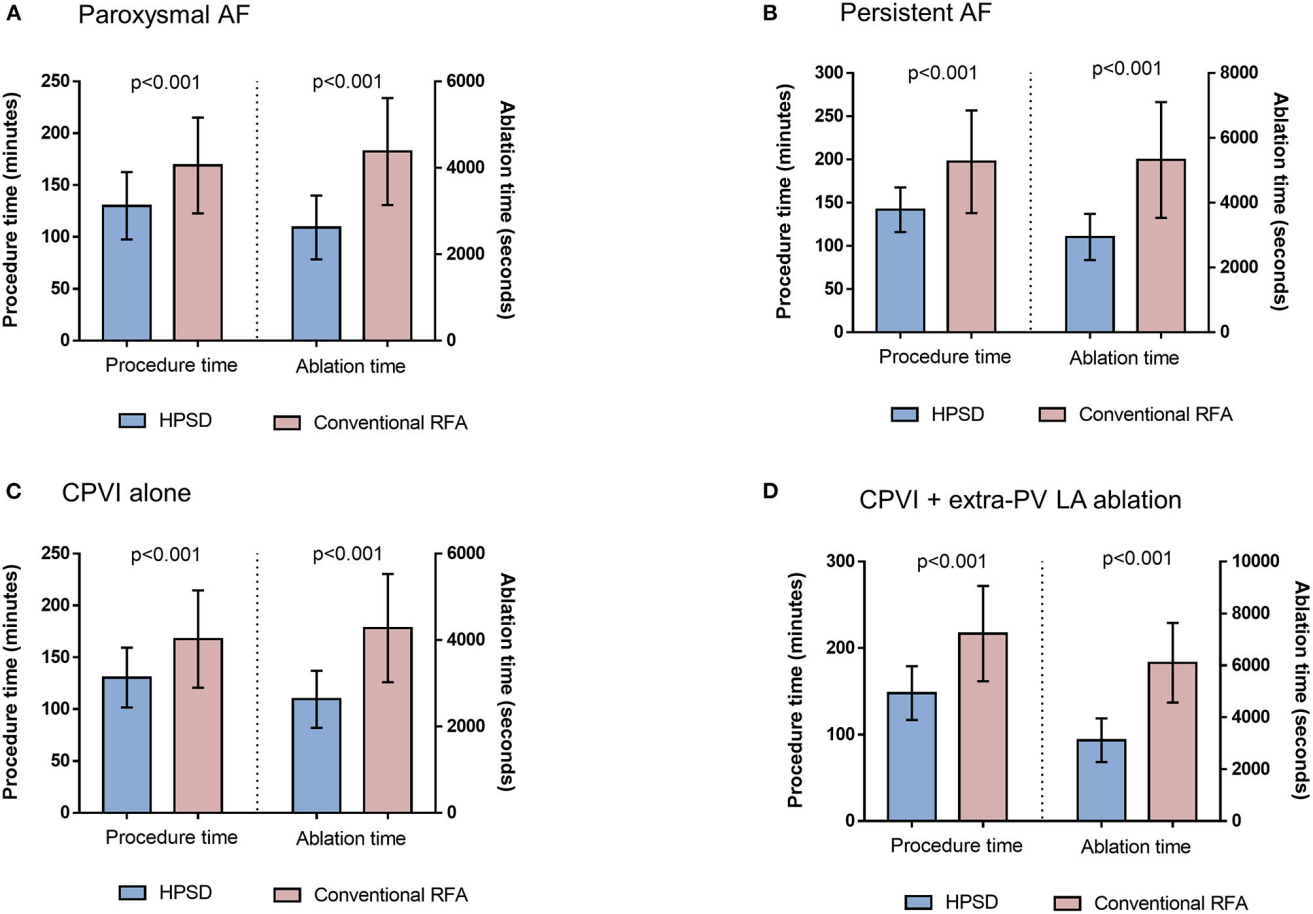
The comparisons of procedure time and ablation time between HPSD and conventional power groups in the patients with paroxysmal AF **(A)**, persistent AF **(B)**, circumferential pulmonary vein isolation (CPVI) alone **(C)**, or additional extra-pulmonary vein (PV) left atrial (LA) ablation **(D)**. HPSD, high-power short-duration radiofrequency ablation; AF, atrial fibrillation; RFA, radiofrequency ablation.

The procedure time (*p* < 0.001) and ablation time (*p* < 0.001) were remarkably shorter in the HPSD group than in the conventional power group (Table 2). In the subgroup analysis according to AF type and ablation lesion sets, the HPSD groups showed significantly reduced procedure and ablation times compared to the conventional power group among paroxysmal AF (*p* < 0.001, Figure 4A), persistent AF (*p* < 0.001, Figure 4B), CPVI alone (*p* < 0.001, Figure 4C), or additional extra-PV LA ablation (*p* < 0.001, Figure 4D). In the multivariate linear regression analysis, the HPSD was independently associated with short procedure time (β = −0.77 [−0.86 to −0.67], *p* < 0.001) and ablation time (β = −33.96 [−36.55 to −31.37], *p* < 0.001, Supplementary Table 2).

## Discussion

### Main Findings

In this single-center, retrospective cohort study, we investigated the difference in the HPSD-AFCA and conventional power AFCA in propensity-score-matched population in terms of their efficacy, safety, and autonomic neural effects. The rhythm outcomes and complication risk of the HPSD-AF ablation were similar to those of the conventional power ablation regardless of the AF type or ablation lesion set. The 3rd month heart rate variability did not differ significantly between the HPSD-AFCA and conventional power AFCA. HPSD-AF ablation is similar to conventional power AF ablation and can significantly shorten the procedure time.

### Unmet Needs in AFCA

Recent clinical data after AFCA have supported the long-term positive clinical effects of AFCA (2, 19–22). Nevertheless, long-term AF recurrence rates are unsatisfactory after this invasive procedure (23). In patients who exhibit sustained AF after AFCA, the long-term prognosis is also worse compared to that in those with a significant reduction in the AF burden (20–22). Various efforts have been made to reduce the long-term recurrence rate after AFCA by applying various lesion sets including atrial substrate ablation (4, 24, 25) as well as identifying prognostic factors useful for patient selection (26, 27). However, the most consistent evidence suggests that a long-lasting CPVI has the greatest effect on the long-term rhythm outcomes, while AF recurrence without a PV reconnection is associated with poorer rhythm outcomes after a repeat ablation because of AF progression or extra-PV foci (8, 13). AFCA outcomes can vary depending on the RF power, catheter design, contact force monitoring, or operator experience (28, 29). In this study, we evaluated the efficacy and safety of the HPSD-AFCA, which facilitates a contiguous lesion formation in areas with a relatively thin atrial wall thickness (10). HPSD-AFCA yielded similar results to conventional power AFCA regardless of a paroxysmal or persistent type, the use of a CPVI alone, or an additional posterior box ablation. In this study, there was a trend for less AT recurrence and rate of cardioversion after recurrence in the HPSD group compared to the conventional power group. So, a future study would be needed to investigate the efficacy of HPSD depending on the type of recurrence and need for cardioversion after recurrence.

### Benefits of HPSD RF Ablation

The efficacy and safety of HPSD AFCA remain controversial and further confirmation is needed. Baher et al. (11) reported similar efficacy of a 50 W HPSD PVI with both contact force and non-contact force catheters as to that of the conventional power PVI in 687 patients with AF. Kottmaier et al. (12) reported superior rhythm outcomes of a 70W HPSD PVI in 197 AFCAs using a flexible tip mesh-type catheter. In this study, there was no difference in the efficacy or safety of a 50–60 W HPSD ablation as compared to a conventional power AFCA. We also proved that the autonomic denervation effect, which requires a transmural lesion formation of the subepicardium (17), was equivalent between a HPSD ablation and the conventional methods. The greatest advantage of the HPSD ablation is the reduction in procedure time. Nevertheless, a one-shot balloon technology, very high power RF ablation, and pulse-field ablation, which also shorten the ablation time, are in continuous development, so a future comparative evaluation of the HPSD AFCA with these techniques is warranted (30–33).

### Potential Adverse Effects of HPSD AFCA

The biggest concern of HPSD AFCA is the need to verify the risk of adverse effects. In particular, cauterization of thin atrial walls with high power RF energy poses risks of steam pops, cardiac tamponade, and collateral damage (10, 11, 34). Although HPSD-AFCA did not raise the risk of major complications in this or previous studies, special attention should be paid to micro-steam pops, impedance changes, and over-heating during the RF energy delivery. During an LA posterior wall ablation, damage to the esophagus or posterior mediastinal structures can occur. There is still no evidence that HPSD ablation increases esophageal damage and it is possible to reduce deep tissue injury by reducing the irrigation flow (10, 11, 34). In this study, all patients were monitored for esophageal temperature during the posterior LA ablation with an RF power of 40~50 W for <10 s. Because the lesion formation is also affected by the catheter design, catheter-tissue contact surface, and current density, it is not reasonable to use the same indicator in catheters with different designs during HPSD ablation; more experience and research are required (35). In rare cases, a small amount of char formation was observed during ablation with a HPSD with a mesh-type flexible tip catheter. Therefore, sheath irrigation is recommended after completing a single-sided CPVI. In the future, additional research should explore how to control the duration and power of the HPSD ablation while monitoring the LA wall thickness (36).

### Limitations

Several limitations of this study should be recognized. First, since this study was reported from a single center and involved a relatively small number of patients, the results from this study cannot be completely applied to all patients with an HPSD-AFCA. However, there was also an advantage of this single-center cohort in that the ablation and rhythm follow-up protocols were consistent. Second, although we performed regular rhythm follow-up visits in all included patients, the AF burden could not be exactly investigated by Holter monitoring. We could not conduct HRV analyses in all included patients, because some of them conducted follow-up Holter from other hospitals. Third, since the HPSD ablation group underwent AFCA with a non-contact force mesh-type flexible tip ablation catheter, we monitored the RF delivery duration but not the other quantitative units such as the ablation index or lesion index. Because we analyzed the long-term registry data, we could not compare total amounts of RF energy or the first pass PVI rates in this study (37). Fourth, the prescription rates of AAD were relatively high and uncontrolled; thus, medications may have affected the rhythm outcomes of the AFCA in both groups. Although the proportions of beta-blocker users were not statistically different between the two groups, beta-blocker may affect RR interval variability and its spectral components (38). Fifth, despite the propensity-matched comparison, there was an evolution of the mapping system, and the catheters and the ablation technique do not remain the same during the long-term period of patient inclusion. Sixth, although similar results were reported in the previous studies (39, 40), this study reported outcomes including autonomic neural effects of HPSD in the large population cohort with propensity score matching. Finally, this study was an observational study and even propensity score matching could not compensate for all confounding factors such as center experience and advanced ablation technology might affect the outcome of this study. However, this study included a large real-world population and reported results after multivariate and subgroup analysis in circumstances of lacking data from the randomized controlled trial study.

## Conclusions

HPSD-AFCA notably reduced the procedure time with similar rhythm outcomes, complication rate, and influence on autonomic function as compared to the conventional power AFCA, irrespective of the AF type or ablation lesion set.

## Data Availability Statement

The datasets presented in this article are not readily available because IRB approval is mandated. Requests to access the datasets should be directed to Hui-Nam Pak, hnpak@yuhs.ac.

## Ethics Statement

The studies involving human participants were reviewed and approved by the Institutional Review Board of the Yonsei University Health System. The patients/participants provided their written informed consent to participate in this study.

## Author Contributions

H-NP designed the study, analyzed and interpreted data, drafted the manuscript, and did final approval of the manuscript submitted. J-WP designed the study, analyzed and interpreted data, and drafted the manuscript. S-YY analyzed and interpreted data. MK, HY, T-HK, and J-SU contributed to acquiring the patients' clinical data. BJ and M-HL revised the manuscript for important intellectual content. All authors contributed to the article and approved the submitted version.

## Funding

This work was supported by grants (HI19C0114) and (HI21C0011) from the Ministry of Health and Welfare and a grant (NRF-2020R1A2B01001695) from the Basic Science Research Program run by the National Research Foundation of Korea (NRF), which is funded by the Ministry of Science, ICT & Future Planning (MSIP).

## Conflict of Interest

The authors declare that the research was conducted in the absence of any commercial or financial relationships that could be construed as a potential conflict of interest.

## Publisher's Note

All claims expressed in this article are solely those of the authors and do not necessarily represent those of their affiliated organizations, or those of the publisher, the editors and the reviewers. Any product that may be evaluated in this article, or claim that may be made by its manufacturer, is not guaranteed or endorsed by the publisher.

## Abbreviations

AAD: antiarrhythmic drug
AFCA: AF catheter ablation
AT: atrial tachycardia
ConvP: conventional power
CPVI: circumferential pulmonary vein isolation
CTI: cavotricuspid isthmus
HPSD: high power short duration
HRV: heart rate variability
LA: left atrium.
