# Supplementary material for: Efficacy and Safety of High-Power Short-Duration Radiofrequency Catheter Ablation of Atrial Fibrillation
Source: Front Cardiovasc Med. 2021 Oct 7;8:709585. doi: 10.3389/fcvm.2021.709585 (PMC8530188; doi:10.3389/fcvm.2021.709585)
Supplement: Supplementary file 1 [file Data_Sheet_1.docx]

**Supplementary Table 1. Standardized mean differences (SMD) of variables before and after propensity-score matching (PSM)**

|  | **Pre-PSM SMD** | **Post-PSM SMD** |
| --- | --- | --- |
| Age | 0.079 | 0.010 |
| Male | 0.002 | 0.007 |
| Paroxysmal atrial fibrillation | 0.268 | 0.028 |
| Body mass index | 0.102 | 0.023 |
| Congestive heart failure | 0.323 | 0.020 |
| Hypertension | 0.031 | 0.004 |
| Diabetes mellitus | 0.048 | 0.017 |
| Stroke/transient ischemic attack | 0.016 | 0.003 |
| Vascular disease | 0.255 | 0.025 |
| CHA_2_DS_2_-VASc score | 0.077 | 0.008 |
| Left atrial dimension | 0.254 | 0.037 |
| Cavotricuspid isthmus ablation | 0.490 | 0.009 |
| Extra-pulmonary vein left atrial ablation | 0.084 | 0.012 |

**Supplementary Table 2. Baseline and ablation variables associated with procedure and ablation time**

|  | **Univariate*** | | **Multivariate*** | |
| --- | --- | --- | --- | --- |
|  | **β (95% CI)** | **p-value** | **β (95% CI)** | **p-value** |
| Procedure time (hour)* |  |  |  |  |
| Paroxysmal AF | -0.395 (-0.492 to -0.299) | <0.001 | -0.132 (-0.236 to -0.028) | 0.013 |
| CHF | 0.139 (0.021 to 0.258) | 0.021 | -0.114 (-0.241 to 0.014) | 0.081 |
| LA dimension | 0.029 (0.021 to 0.306) | <0.001 | 0.008 (-0.001 to 0.016) | 0.075 |
| LVEF | -0.007 (-0.012 to -0.001) | 0.014 | 0.002 (-0.004 to 0.007) | 0.537 |
| E/Em | 0.009 (-0.002 to 0.021) | 0.123 | 0.003 (-0.007 to 0.014) | 0.540 |
| LVEDD | 0.010 (0.000 to 0.021) | 0.051 | 0.002 (-0.008 to 0.013) | 0.066 |
| CT volume index | 0.006 (0.005 to 0.008) | <0.001 | 0.002 (0.000 to 0.004) | 0.024 |
| CTI ablation | 1.170 (0.753 to 1.587) | <0.001 | 1.071 (0.710 to 1.433) | <0.001 |
| Extra-PV LA ablation | 0.694 (0.590 to 0.797) | <0.001 | 0.560 (0.452 to 0.668) | <0.001 |
| HPSD | -0.762 (-0.867 to -0.658) | <0.001 | -0.767 (-0.863 to -0.670) | <0.001 |
| Ablation time (min)* |  |  |  |  |
| Male | 3.976 (0.510 to 7.442) | 0.025 | 4.248 (1.438 to 7.058) | 0.003 |
| Paroxysmal AF | -12.690 (-15.698 to -9.682) | <0.001 | -1.614 (-4.346 to 1.118) | 0.247 |
| CHF | 4.060 (0.367 to 7.754) | 0.031 | -2.285 (-5.532 to 0.962) | 0.168 |
| Stroke/TIA | 3.689 (-0.987 to 8.364) | 0.122 | 1.973 (-1.513 to 5.458) | 0.267 |
| Vascular disease | 7.405 (0.337 to 14.472) | 0.040 | 3.527 (-1.623 to 8.677) | 0.179 |
| LA dimension | 0.991 (0.756 to 1.227) | <0.001 | 0.375 (0.152 to 0.598) | 0.001 |
| LVEF | -0.299 (-0.462 to -0.135) | <0.001 | 0.072 (-0.076 to 0.220) | 0.342 |
| LVEDD | 0.406 (0.084 to 0.727) | 0.014 | -0.027 (-0.309 to 0.255) | 0.851 |
| CT volume index | 0.207 (0.150 to 0.264) | <0.001 | 0.099 (0.048 to 0.151) | <0.001 |
| CTI ablation | 28.616 (15.543 to 41.689) | <0.001 | 24.807 (15.208 to 34.407) | <0.001 |
| Extra-PV LA ablation | 25.042 (21.892 to 28.192) | <0.001 | 21.831 (18.945 to 24.716) | <0.001 |
| HPSD | -33.488 (-36.468 to -30.507) | <0.001 | -33.957 (-36.546 to -31.369) | <0.001 |

* Units of procedure time and ablation time are hours and minutes respectively. Univariate analysis was performed using the variables including age, male sex, paroxysmal AF, body mass index, CHF, hypertension, diabetes mellitus, stroke/TIA, vascular disease, CHA2DS2-VASc score, LA dimension, LVEF, E/Em, LVEDD, CT volume index, CTI ablation, extra-PV LA ablation, HPSD. The variables with p-value ≤0.2 in the univariate analysis were included in the multivariate analysis. AF=atrial fibrillation, CHF=congestive heart failure, LA=left atrium, LVEF=left ventricular ejection fraction, E/Em=mitral inflow velocity/mitral annulus tissue velocity, LVEDD=left ventricular end diastolic dimension, CT=computed tomography, CTI=cavotricuspid isthmus, PV; pulmonary vein, HPSD; high power short duration, TIA; transient ischemic attack
